# A Colony for Defectives

**Published:** 1920-10-23

**Authors:** 


### A Colony for Defectives.

Middlesex County Council's Committee for the care of Mentally Defectives has concluded that it would, from an administrative and economic point of view, be of advantage to co-operate with the local education authorities in the county in providing accommodation for mentally deficient children between the ages of seven and sixteen. The County Architect has accordingly collected particulars during the last year of some forty sites for the establishment of a colony, and the Committee favours one at Harefield Grove, which is practically next to the Harefield Park Sanatorium. The estate comprises 381 acres, and includes two residences, thirteen cottages, three farms with good buildings, and various other buildings which could be adapted for defectives. The price asked is £50,000. It is computed that about 200 high-grade defectives could at once be accommodated without any additional building, and that their labour could be utilised in erecting other buildings. The Committee believes that sufficiently substantial buildings of the semi-permanent type could be erected by the best of the inmates under skilled supervision, and the colony could be added to bit by bit to meet requirements as they arise without very much capital outlay in one year. Mental defectives of the highest grades are capable, under proper supervision and training, of being employed in the laundry, power-house, bakehouse, at various agricultural operations on the land, and at various industrial operations in workshops. The labour of the defectives on the land could be utilised to supply both institutions with dairy and market-garden produce. Though the capital cost will be borne in the first instance by the County Council, one half will ultimately be paid by the Board of Control.

